# MAIA and Humphrey Perimetry Differ in Their Estimation of Homonymous Visual Field Defects

**DOI:** 10.1167/tvst.13.11.15

**Published:** 2024-11-14

**Authors:** Bryan V. Redmond, Berkeley K. Fahrenthold, Jingyi Yang, Elizabeth L. Saionz, Matthew R. Cavanaugh, Krystel R. Huxlin

**Affiliations:** 1 Flaum Eye Institute and Center for Visual Science, University of Rochester Medical Center, Rochester, New York

**Keywords:** hemianiopia, cortical blindness, perimetry, vision restoration, reliability

## Abstract

**Purpose:**

To assess whether the Humphrey Visual Field Analyzer (HFA) and the Macular Integrity Assessment instrument (MAIA) provide equivalent estimates of visual deficit size, severity, and progression in cortically blinded participants.

**Methods:**

Reliable, monocular 10-2 HFA and MAIA fields were collected at baseline, and after a blind-field training intervention (*n* = 54) or no intervention (*n* = 6) in adult participants with occipital strokes. Binocular HFA and MAIA mean sensitivities (MS) were first computed, before creating binocular maps of visual sensitivity for each perimetry system to calculate deficit areas, using a unitary, published, less than 10 dB criterion to define blindness. We contrasted HFA/MAIA MS and deficit area at individual study visits, together with change in these measures between visits.

**Results:**

At individual visits, MS and deficit areas were well-correlated, but there were systematic differences between machines, greater for the intact than impaired hemifields, with the MAIA overestimating areas of visual deficit relative to the HFA. Between visits, the two perimeters’ assessment of change in MS was correlated, but change in the deficit area was not, despite good test reliability.

**Conclusions:**

Reliable HFA and MAIA tests produce well-correlated but systematically offset estimates of visual sensitivity and deficit area in patients with homonymous field defects, which can impact assessment of progression.

**Translational Relevance:**

Cortically blinded patients exhibited comparable changes in sensitivity (spontaneous and training induced) when assessed using Humphrey and fundus-controlled MAIA perimetry. However, the photopic vs. mesopic nature of these tests impacted participant performance and suggests that custom, machine-specific criteria are needed to comparably define blindness on both systems.

## Introduction

Cortically induced blindness (CB) is a debilitating consequence of damage to the occipital lobe of the brain, with unilateral lesions causing contralesional homonymous visual defects, ranging from a small scotoma to a full hemifield of visual impairment. Although CB has been considered untreatable traditionally, a growing body of evidence suggests that it may be neither intractable nor homogenously blind.^1^^–^^3^ As such, collecting high-quality visual perimetry is increasingly critical to the management of CB.^4^

Among perimetry systems,^5^^–^^7^ automated perimeters tend to be preferred clinically for rapid diagnosis and quantification of visual field defects.^8^ The Humphrey Field Analyzer (HFA) is the most commonly used automated perimeter in clinical settings and the most widely used for clinical trials involving visual field assessments in the United States.^9^^,^^10^ The HFA tests patients using a Goldmann size III stimulus, with maximum brightness of 10,000 asb, on a background in the photopic range (10 cd/m^2^), making it an increment detection task. It monitors fixation by tracking the pupil.

In contrast, microperimeters, which combine fundus imaging of the retina with gaze-contingent stimulus presentation, have introduced a more precise method for assessing gaze and thus, visual field defects. One such device, available in many clinics in the United States, is the Macular Integrity Assessment instrument (MAIA). It images and tracks the fovea, the true anatomical point of fixation, during testing; it then adjusts presentation of a spot target to be detected by adjusting for foveal movement during fixation.^11^ Thus, fundus-controlled perimetry represents a more rigorous approach for assessing the visual field than any other perimetry systems, including the HFA, which uses pupil tracking and no automated gaze-shift compensation.^12^^–^^14^ In fact, fundus-controlled perimetry was used to counter NovaVision's claim of blind-field border shifts in CB patients after computer-based training, showing that these shifts disappeared if patients were tested with gaze-contingent perimetry.^15^^,^^16^ However, a subsequent article countered, stating that measures of training-induced improvements vary depending on the perimeter used, and that perimeters with differing stimuli and tasks can estimate deficits differently.^17^ Indeed, in contrast with the HFA, the MAIA presents a Goldmann size III stimulus with a maximum brightness of 1000 asb, on a background in the mesopic range (1.27 cd/m^2^).

A number of visual restoration studies in CB have since claimed that visual discrimination training can induce substantial changes in the HFA-defined blind field, including border shifts and decreased deficit area, whereas no intervention results in small spontaneous changes and in some cases, increased deficit area.^18^^–^^20^ Thus, patients with CB are ideally suited to address the question of whether only the HFA can detect such visual field changes—either owing to the nature of its task, or because it cannot compensate for tiny fixation shifts during test performance. Alternatively, it is possible that both the HFA and fundus-controlled perimetry can detect clinically significant changes in visual defects, but that differences in the tests’ conditions cause large differences in magnitude or location/distribution of these changes.

To answer these questions, we conducted a retrospective analysis comparing the size and severity of visual field defects in CB, measured over an identical region of the central visual field by Humphrey and MAIA perimeters in a substantial cohort of occipital stroke patients with CB. Most of the patients underwent computer-based visual discrimination training between two timepoints, while a smaller group remaining untrained. This study design allowed us to maximize the range of changes in the size and severity of visual field defects over which to compare the two devices’ performance side by side.

## Methods

### Study Design

A retrospective analysis was performed of occipital stroke patients with CB enrolled in two research studies at the University of Rochester (ClinicalTrials.gov Identifiers: NCT04798924, NCT05098236). The studies were approved by the University of Rochester's Institutional Review Board and adhered to the tenets of the Declaration of Helsinki. Written, informed consent was obtained from all participants.

**Figure 1.**
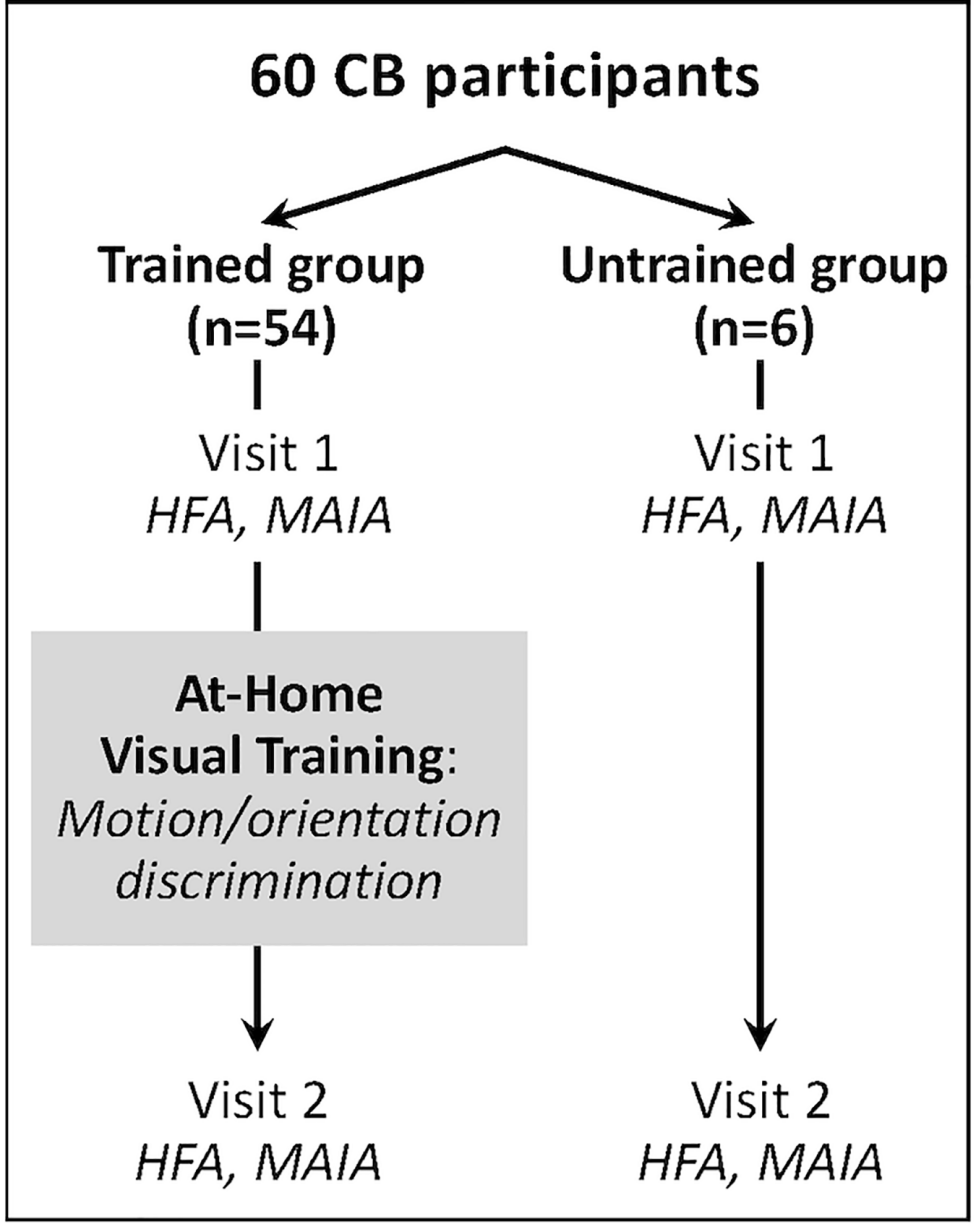
Study design. Participants selected for this study performed two sets of HFA and Macular Integrity Assessment (MAIA) perimetry tests: the first at visit 1, after which participants either performed at-home visual training or remained untrained until visit 2, when both HFA and MAIA perimetry were repeated.

Participants were selected for the present analysis if they underwent HFA and MAIA perimetry at two different timepoints (labeled visits 1 and 2), with all tests meeting reliability criteria (<20% false positives, false negatives, and fixation losses). In 54 patients, the two timepoints straddled a visual training intervention that involved either motion discrimination using random dot or Gabor stimuli, or orientation discrimination of Gabor stimuli.^1^^,^^20^^–^^25^ Six of the participants remained untrained. The mean interval between visits 1 and 2 was 7.2 ± 6.0 months (range, 0.5–36.9 months) for trained participants and 7.0 ± 5.3 months (range, 2.6–16.6 months) for untrained participants (Table 1, Fig. 1), values that were not statistically different (unpaired Student's *t* test: t_58_ = −0.1; *P* = 0.91; 95% confidence interval [CI], ±5.66).

**Table 1.** Participant Demographics

Patient	Gender	Age (years)	Affected Hemifield	Visit 1 Time Since Stroke (months)	Visit 2 Time Since Stroke (months)	Training Type	Data Published
CB1	Female	26	Bilateral	7.5	12.77	Ori, Motion	4
CB2	Female	27	Left	29.9	42.9	Motion	3
CB3	Female	27	Right	3.5	12.67	Motion	5
CB4	Male	34	Left	1.9	6.27	Motion	5
CB5	Male	35	Left	4.3	6.83	Motion	5
CB6	Female	39	Left	3.0	6.8	Motion	2
CB7	Male	39	Left	4.0	11.87	Motion	5
CB8	Male	39	Left	1.4	6.6	Motion	5
CB9	Male	42	Right	2.7	14.83	Ori, Motion	4
CB10	Female	43	Left	3.1	6.2	Motion	5
CB11	Male	43	Left	2.8	6.1	Motion	5
CB12	Female	44	Left	2.6	6.3	Motion	2
CB13	Male	47	Bilateral	28.5	65.47	Motion	1, 3
CB14	Male	47	Right	5.1	12.97	Motion	5
CB15	Male	48	Right	4.8	6.17	Motion	5
CB16	Female	48	Right	4.6	12.37	Motion	5
CB17	Male	49	Right	1.4	9.8	Motion	3
CB18	Female	49	Right	3.2	12.03	Motion	5
CB19	Male	50	Left	5.03	5.53	Motion	5
CB20	Male	51	Right	3.5	6.2	Motion	5
CB21	Male	51	Left	3.0	12.97	Motion	5
CB22	Female	52	Left	52.3	59.33	Motion	1
CB23	Male	52	Left	13.2	30.93	Ori, Motion	4
CB24	Female	52	Right	4.4	12.43	Motion	5
CB25	Female	53	Right	3.83	5.86	Motion	5
CB26	Female	54	Left	30.4	48.73	Motion	1
CB27	Male	54	Left	2.57	5.97	Motion	5
CB28	Male	57	Left	1.7	7.3	Motion	2
CB29	Male	57	Left	2.8	6.27	Motion	5
CB30	Male	57	Right	3.2	12.83	Motion	5
CB31	Male	57	Left	3.7	6.3	Untrained	5
CB32	Female	57	Right	4.4	7.17	Motion	5
CB33	Female	59	Right	4.0	12.37	Motion	5
CB34	Male	60	Left	3.0	8.2	Ori, Motion	4
CB35	Male	60	Bilateral	1.8	6.93	Motion	4
CB36	Male	60	Right	121.4	126.5	Ori	4
CB37	Female	60	Left	1.3	6.53	Motion	5
CB38	Male	61	Left	1.7	5.67	Motion	2
CB39	Male	61	Right	3.3	7.03	Ori	4
CB40	Female	61	Left	2.7	16.23	Ori	4
CB41	Female	61	Right	6.3	12.17	Ori, Motion	4
CB42	Female	62	Left	24.7	29.4	Ori, motion	4
CB43	Male	65	Right	22.6	32.1	Untrained	2
CB44	Male	65	Right	37.2	48.97	Motion	4
CB45	Female	66	Right	2.6	6.93	Ori, Motion	2
CB46	Female	68	Left	26.7	43.87	Motion	1
CB47	Female	68	Right	1.6	6.47	Motion	4
CB48	Male	68	Left	2.3	7.53	Motion	4
CB49	Female	68	Right	4.6	6.93	Motion	5
CB50	Male	68	Left	1.97	6.37	Motion	5
CB51	Male	69	Right	2.3	7.47	Untrained	2
CB52	Male	69	Right	1.4	5.87	Untrained	2
CB53	Male	70	Right	2.1	6.5	Motion	2
CB54	Male	70	Left	0.6	17.2	Untrained	2
CB55	Male	70	Right	1.6	6.7	Motion	5
CB56	Male	72	Right	15.6	20.27	Ori	4
CB57	Male	74	Right	1.1	7.63	Motion	2
CB58	Male	74	Left	10.2	28.87	Motion	3
CB59	Male	74	Left	2.3	7.2	Motion	5
CB60	Male	77	Left	2.6	6.03	Untrained	2

Ori, orientation discrimination task; Motion, motion discrimination task.

Data Published codes: 1 = 16; 2 = 1; 3 = 17; 4 = 18; and 5 = unpublished data from ongoing clinical trial NCT04798924

### Participants

Data were obtained from 60 occipital stroke patients (Table 1), with a mean age of 55.7 ± 12.6 years; including 38 male and 22 female patients; and 3 bilateral deficits, 30 left-sided deficits, and 27 right-sided deficits. Time after stroke at initial testing was 9.3 ± 18.0 months (median, 3.2 months; interquartile range, 2.7 months). Upon initial screening into their respective studies (NCT04798924, NCT05098236) stroke location was verified by structural imaging when available (Supplementary Fig. S1). Participants were included if they were residents of the United States or Canada, had magnetic resonance imaging or computed tomography scans showing stroke damage to occipital cortex, and were deemed to have sufficiently intact motor and cognitive functions on their medical records to complete our tasks (computerized testing and training, as well as perimetry). Exclusion criteria included past or present ocular and neurological disease, corrected vision worse than 20/40 in either eye, damage to the dorsal lateral geniculate nucleus, current use of neuroactive drugs, history of traumatic brain injury or whole brain degenerative process, and cognitive or seizure disorders.

### Humphrey Visual Field Analyzer (HFA) Testing

**Figure 2.**
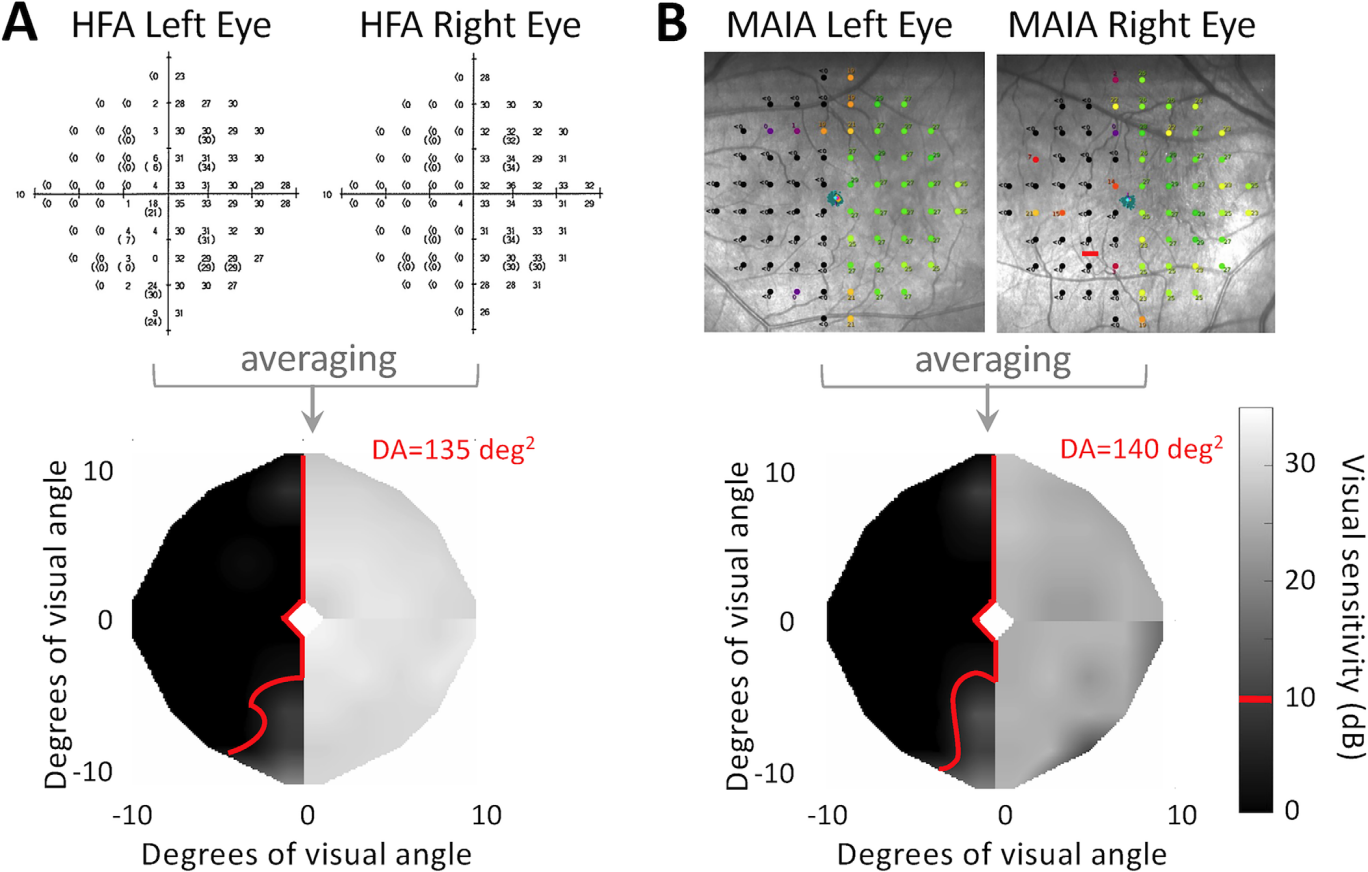
Method for creating binocular maps from raw perimetry data, illustrated for one patient—CB07—at visit 1. (A) Sensitivity data at each test point obtained during monocular HFA tests are averaged together between the eyes before undergoing a two-dimensional interpolation to generate a single binocular map of luminance sensitivity across the central 10° of the visual field. As foveal values are not assessed by the MAIA, we excluded foveal sensitivity from the HFA maps, represented as a 2° × 2° diamond at the center of each map. Finally, the Social Security Administration definition of blindness, less than10 dB of sensitivity, was used to compute the deficit area (DA, outlined in red along the vertical meridian) in each map. (B) Comparable methods as in (A) for MAIA tests, with monocular MAIA fields flipped vertically before interpolation to account for their presentation. Note the slightly larger MAIA deficit area compared with the HFA in (A).

Monocular HFAs were collected on the Humphrey Field Analyzer II (i750-30267, version 5.1.1; Carl Zeiss Meditec, Jena, Germany) by trained ophthalmic technicians. Visual acuity was corrected to 20/40 or better in each eye. Patients were adapted to ambient (dim light) conditions for 10 minutes before starting the test. Fixation was controlled using the Gaze/Blind Spot automatic settings and monitored by the technician. Although the HFA can test a wider field of view, the 10-2 test pattern, consisting of 68 locations with a 2° sampling resolution and covering the central ±10° of vision, was used because of its critical role in daily functioning, and because it exactly matched the MAIA's field of view (Fig. 2A). Testing was performed in a dimly lit room and took 5.7 to 17.1 minutes per eye (Supplementary Fig. S2), with a break between eyes. White, Goldmann size III stimuli (4 mm^2^ area) were used, testing light intensities from 10,000 asb to 0.1 asb, presented on a background with luminance of 10 cd/m^2^. Patients were asked to respond with a clicker each time they detected a spot of light. Notably, during the 10-2 testing pattern, the HFA selectively retests 10 locations twice, generating a short-term fluctuation value, reflective of the variability in threshold at specific locations.^26^ The HFA provides two sensitivities for those test sites, which we averaged together so that each location was represented by a single number.

In most cases, the full threshold strategy was used, which changes the intensity of the stimulus in 4-dB increments until the patient's response changes from first presentation (from perceived to not perceived or vice versa). After this reversal, step size changes to 2 dB at that location. Another change in the patient's response sets the threshold for that location. Four fields from four patients were obtained using the Swedish Interactive Threshold Algorithm (SITA) standard threshold strategy. The SITA strategy operates on the assumption that the sensitivity at a specific test location is likely to be normal. Consequently, it requires fewer trials to verify normal sensitivity but more trials (and time) to confirm sensitivity levels in the intermediate range (e.g., 10–20 dB), as opposed to using a uniform prior probability distribution, as is done by the full threshold strategy.^27^^,^^28^ Fields collected using the SITA standard threshold strategy did not include short-term fluctuation values. These patients were removed from short-term fluctuation and testing time analyses, but we should note that no significant difference was reported in prior comparison of full threshold vs. SITA fields collected in occipital stroke patients.^29^ However, patients were excluded from the present analysis if they scored greater than 20% on any one of three test quality metrics automatically computed by the HFA: false positives (when a patient responds but no stimulus was present), false negatives (when a patient does not respond to a stimulus they should have detected), and fixation losses (responding to a stimulus located within the anatomical blind spot).

### Macular Integrity Assessment (MAIA) Perimetry

Monocular visual fields (Fig. 2B) were collected in a dark room using a MAIA perimeter (0074 version 2.5.1, CenterVue), with the same 10-2 testing pattern and white, Goldmann size III stimulus (4 mm^2^ area) as the HFA and a 4-2 full threshold strategy, with stimulus detection likewise indicated with a hand-held clicker. Patients were adapted to dark room lighting conditions for 10 minutes before starting the test, which then took 7.3 to 7.6 minutes per eye (Supplementary Fig. S2). Among other notable differences relative to Humphrey perimetry is the MAIA's background luminance of 1.27 cd/m^2^ and testing light intensities range from 1000 asb to 0.1 asb. Additionally, sensitivity at fixation and the fovea is not assessed in the MAIA. Fixation control also differs from the Humphrey, with retinal location and fixational stability measured at 25 Hz. In addition to reporting fixation losses during the test, fixation locations during MAIA testing are plotted atop the scanning laser ophthalmoscope image of the retina and used to compute 63% and 95% bivariate contour ellipse areas (BCEAs), which encompass fixation locations based on the standard deviations of the horizontal and vertical eye positions.^30^ Because it covers the largest proportion of fixations during the examination, the 95% BCEA (BCEA_95_) was used for the present analyses.

### Outcome Measures

Both the Humphrey and MAIA compute a summary statistic of the patient's visual performance across all 68 test locations. The Humphrey reports the mean deviation (MD), indicating the amount by which the tested field deviates from a normative database, accounting for patient age and the deviation from normal field measurements at each testing location.^27^ A normal MD is 0 dB; a negative MD indicates impaired vision. The MAIA summary statistic is an average threshold for detection sensitivity across all 68 test locations in each eye. This mean sensitivity (MS) is not age corrected or normalized, with an MS of 0 dB denoting blindness across the entire measured field. However, we note that a 0 dB MAIA MS is not necessarily identical to a 0 dB HFA MS (nor is 0 dB at a given testing location), because the luminance of the background and stimuli for each device differs. All patients in the present study generated positive MS values, albeit reduced compared with those that would be obtained in visually intact participants.

To compare the two perimeters directly and bypass the Humphrey's proprietary computation of MD, we calculated an MS for each monocular HFA (excluding the reported foveal sensitivity, since this was not reported by the MAIA), to match the MS computed by the MAIA. Given the homonymous nature of the CB field defect, monocular HFA MS and MAIA MS values were then averaged between the two eyes, generating a single value for each patient at each timepoint. The binocular MS was thus our primary outcome measure for both perimetry systems.

Our secondary outcome measure was the deficit area within the central ±10° of the visual field. To quantify this, we used a previously described,^18^ custom program in Matlab (MathWorks Inc., Natick, MA) to create binocular, interpolated maps of visual sensitivity for each perimeter, in each participant and study visit (Fig. 2). This was done by first averaging luminance sensitivities at each test location for a given machine between the two eyes, before applying a natural neighbor interpolation between test locations. This strategy produced composite visual field maps with a diameter of 20°, an area of approximately 250 deg^2^, and a resolution of 0.1 deg^2^ (Fig. 2). We then used the Social Security Administration's definition of blindness, <10 dB of sensitivity,^31^ as our threshold for defining the deficit area on each composite map, for both perimeters (Fig. 2).

Finally, for HFA-derived outcome measures, we evaluated the relative influence of individual test reliability metrics (fixation losses, false positives, and false negatives) before also assessing the impact of these metrics combined. To do so, we calculated a normalized HFA reliability index (NHRI) as follows:

$$\begin{array}{ccc} & & \;\mathrm{NHRI}=\frac{(\%\;\text{fixation}\;\mathrm{losses}+\%\;\mathrm{false}\;\mathrm{negatives}+\%\;\mathrm{false}\;\mathrm{positives})}{60\%} \end{array}$$

MAIA-derived outcome measures were correlated with the BCEA_95_, whereby monocular BCEA_95_ values were averaged to produce a single value per patient, per visit.

Finally, we note here that our primary objective was not to assess the impact of time or training on perimetry, but rather, to determine whether the two perimeters provided similar parametrization of the deficit at isolated timepoints and between timepoints. As such, the presence of an intervention, and its impact on visual performance, were not considered outcome measures in the present study.

### Statistical Analyses

Two-tailed paired Student's *t* tests were used to compare means across devices and between visits. In all analyses, significance was set at a *P* value of less than 0.05. Linear regression analysis was utilized to characterize the relationship between HFA and MAIA-derived measures, as well as between MS/deficit areas and reliability metrics on each device.

## Results

### Comparing HFA and MAIA Metrics at Baseline (Visit 1)

**Figure 3.**
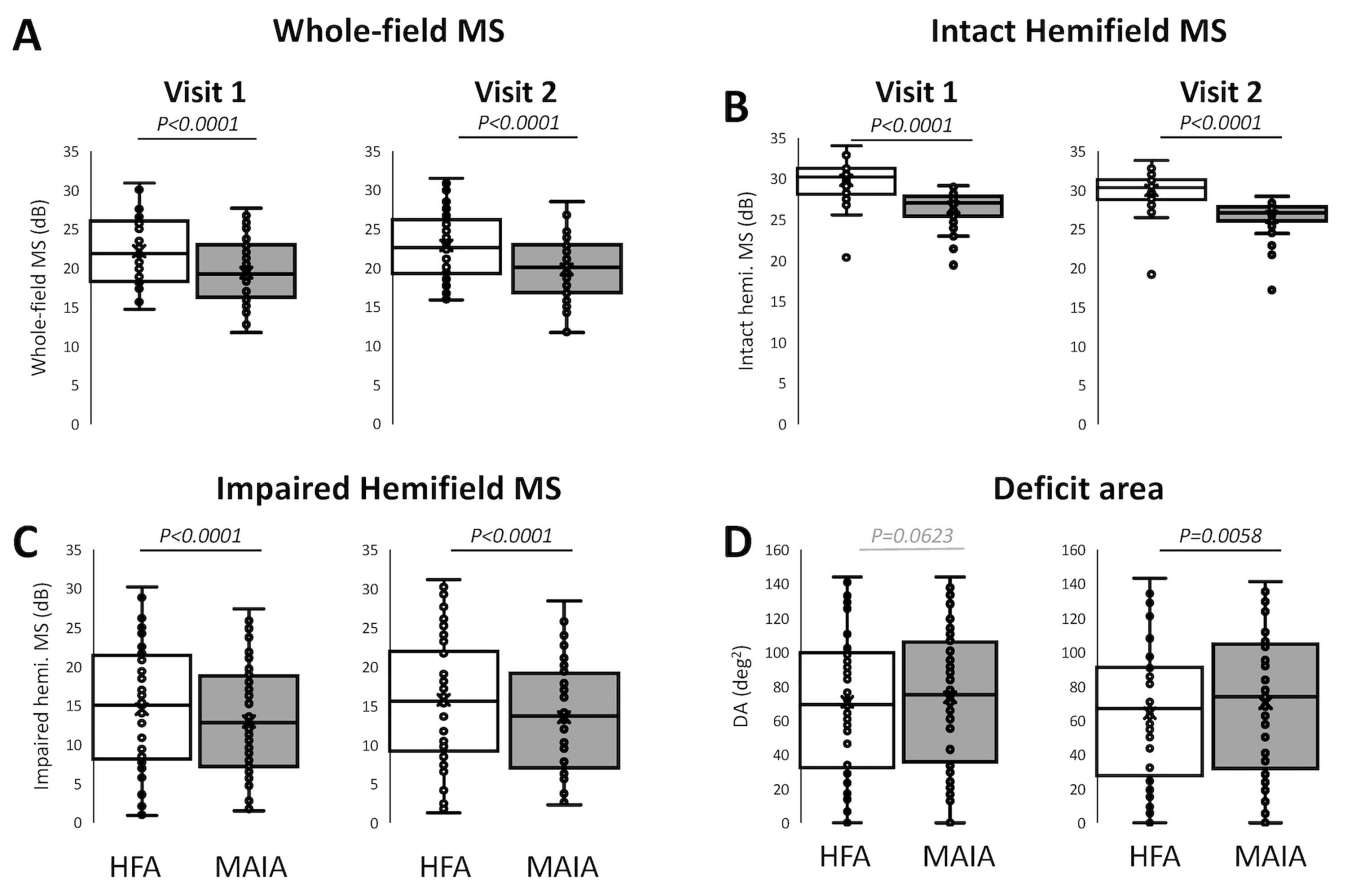
HFA and MAIA-derived MS and deficit areas (DA) at visits 1 and 2. Box and whisker plots of whole-field and hemifield MS and deficit areas at visits 1 and 2. Boxes denote the interquartile range (Q1 to Q3) with the horizontal line in the middle of the box denoting the median. Whiskers at each end represent the minimum and maximum values. Means are indicated with “X”. (A) Visits 1 and 2 HFA and MAIA whole-field MS, with paired Student's *t* tests showing systematic differences between the output of the two perimeters on the same participants (visit 1: t_59_ = 15.72; 95% CI, ±0.35; visit 2: t_59_ = 14.27; 95% CI, ±0.43). (B) Visits 1 and 2 HFA and MAIA intact hemifield MS, with paired Student's *t* tests showing systematic differences between the output of the two perimeters on the same participants (visit 1: t_59_ = 18.42; 95% CI, ±0.36; visit 2: t_59_ = −21.14; 95% CI, ±0.32). (C) Visits 1 and 2 HFA and MAIA impaired hemifield MS, with paired Student's *t* tests showing systematic differences between the output of the two perimeters on the same participants (visit 1: t_59_ = −5.28; 95% CI, ±0.60; visit 2: t_59_ = −6.57; 95% CI, ±0.68). (D) Visits 1 and 2 HFA and MAIA deficit areas (DA) with paired Student's *t* tests showing a significant difference between the two perimeters at visit 2 (visit 1: t_59_ = −1.9; 95% CI, ±3.01; visit 2: t_59_ = −2.86; 95% CI, ±4.19).

At visit 1, the mean HFA testing time, 10.2 ± 2.3 minutes, was slightly longer than the MAIA's (9.6 ± 1.1 minutes; paired *t* test: t_111_ = 3.04, *P* = 0.0026, 95% CI, ±0.41) (Supplementary Fig. S2). MAIA MS averaged 19.4 ± 3.9 dB, significantly lower than HFA MS of 22.2 ± 4.3 dB (Fig. 3A). This difference between MAIA and HFA MS held when restricting the analysis to the intact (Fig. 3B) or impaired hemifields (Fig. 3C), and it was greater (about double) for the intact than impaired hemifields (paired *t* test: t_59_ = −5.54; *P* < 0.0001; 95% CI, 0.63). Finally, the mean HFA deficit area was 70.9 ± 39.9 deg^2^, similar to the mean MAIA deficit area (73.8 ± 39.7 deg^2^) (Fig. 3D). As expected, the deficit area correlated most closely with the impaired hemifield MS, less strongly with the overall MS, and not at all with the intact hemifield MS, regardless of perimeter used (Supplementary Fig. S3). Repeating the analysis of MS monocularly revealed no substantial differences from binocular comparisons (Supplementary Fig. S4), motivating our approach of averaging perimetry data between eyes in this condition in the rest of the study.

### HFA and MAIA Metrics at Visit 2: Sensitivity to Change From Visit 1

At visit 2, the mean HFA testing time (10.4 ± 2.3 minutes) was also slightly longer than the MAIA's (9.8 ± 1.1 minutes; paired *t* test, t_111_ = 3.02; *P* = 0.0031; 95% CI, ±0.44) (Supplementary Fig. S2). Across the entire cohort, the mean HFA and MAIA MS improved to 22.9 ± 4.2 dB and 19.8 ± 4.0 dB, respectively. The change was significant (HFA MS, t_59_ = −4.59; *P* < 0.0001; 95% CI, ±0.31; MAIA MS, t_59_ = −2.22; *P* = 0.030; 95% CI, ±0.33), but the offset between HFA and MAIA MS persisted (Fig. 3A), and the difference between machines continued to be larger in the intact than impaired hemifield (Figs. 3B, 3C). Consistent with an increase in the impaired hemifield's MS relative to visit 1 (HFA, t_59_ = −4.94; *P* < 0.0001; 95% CI, ±0.50; MAIA: t_59_ = −2.54; *P* = 0.0137; 95% CI, ±0.46), deficit area decreased between visits 1 and 2 on both systems (HFA, t_59_ = 3.59; *P* = 0.00067; 95% CI, ±3.62; MAIA, t_59_ =2.72; *P* = 0.0086; 95% CI, ±2.47), with the mean HFA deficit area (64.4 ± 39.4 deg^2^) significantly smaller than MAIA deficit area (70.4 ± 39.0 deg^2^) (Fig. 3D). The intact hemifield exhibited no change in MS measured by either device (HFA, t_59_ = −1.37; *P* = 0.176; 95% CI, ± 0.26; MAIA, t_59_ = −0.81; *P* = 0.421; 95% CI, ±0.27). Repeating this analysis monocularly revealed no substantial differences from binocular comparisons (Supplementary Fig. S4).

**Figure 4.**
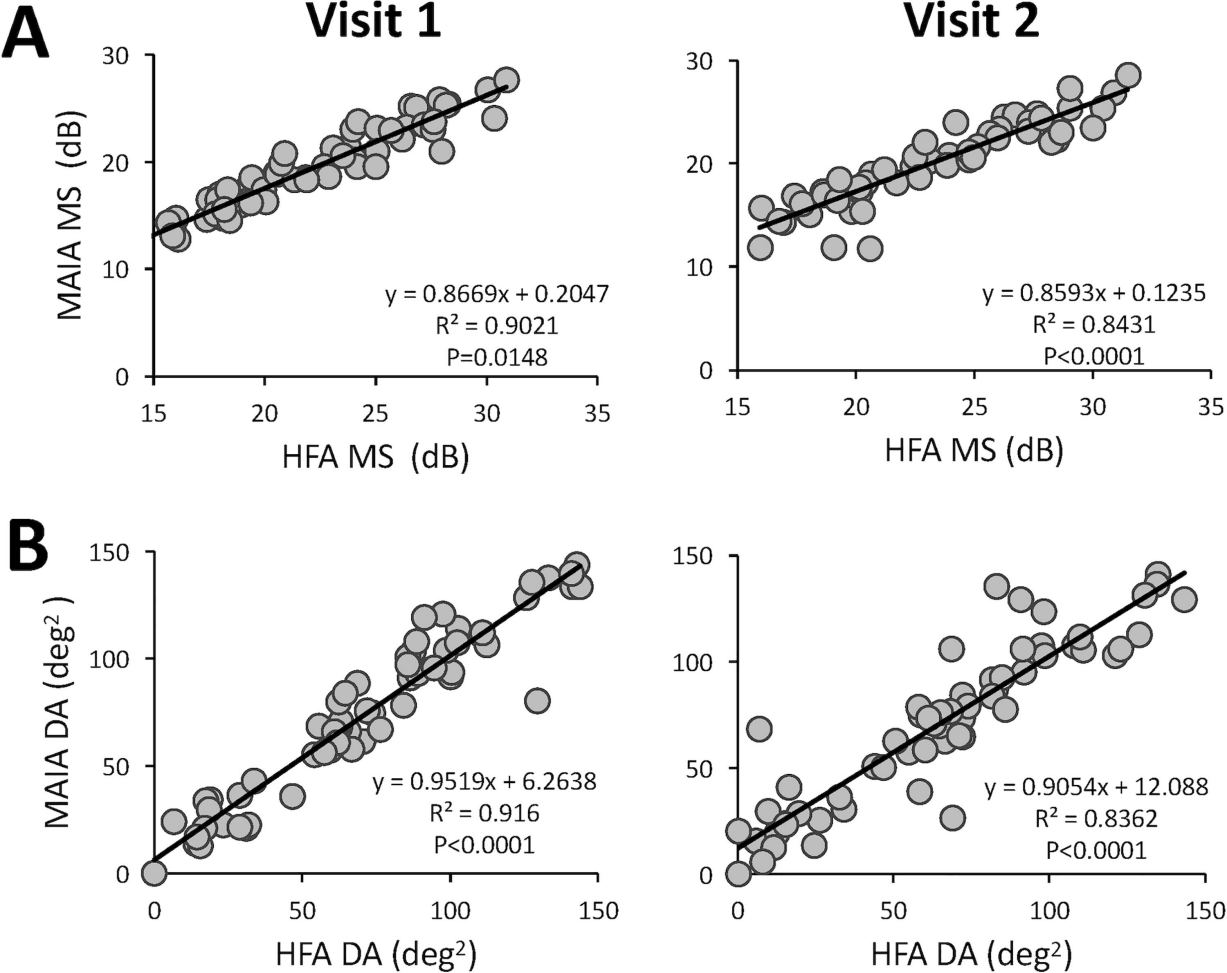
Comparison of HFA and MAIA outputs at single time points. Linear regression analysis of MS and deficit area from the two perimetry systems, with individual circles representing individual patient data points. (A) Visit 1 and visit 2 HFA MS vs. MAIA MS. (B) Visits 1 and 2 HFA vs. MAIA deficit areas (DA). Note extremely tight correlations at visit 1, which weaken by visit 2.

The MS and deficit area were strongly correlated at both visits across machines (Fig. 4). This correlation held within each machine, although as mentioned elsewhere in this article, for visit 1, the deficit area was more strongly correlated with MS in the impaired hemifield than with overall MS or intact hemifield MS at either visit (Supplementary Fig. S3). Nonetheless, there were also marked differences between perimeters. First, regression slopes were always less than 1 and the *y* intercept always positive in Fig. 4, reflecting the fact that MAIA MS and deficit area were systematically lower than HFA MS and deficit area, respectively. Second, R^2^ values were higher at visit 1 than visit 2 (Fig. 4), suggesting that one of the machines was affected more strongly or systematically by the participants’ change in visual performance between tests. To address this possibility, we contrasted between-visit changes in both systems.

**Figure 5.**
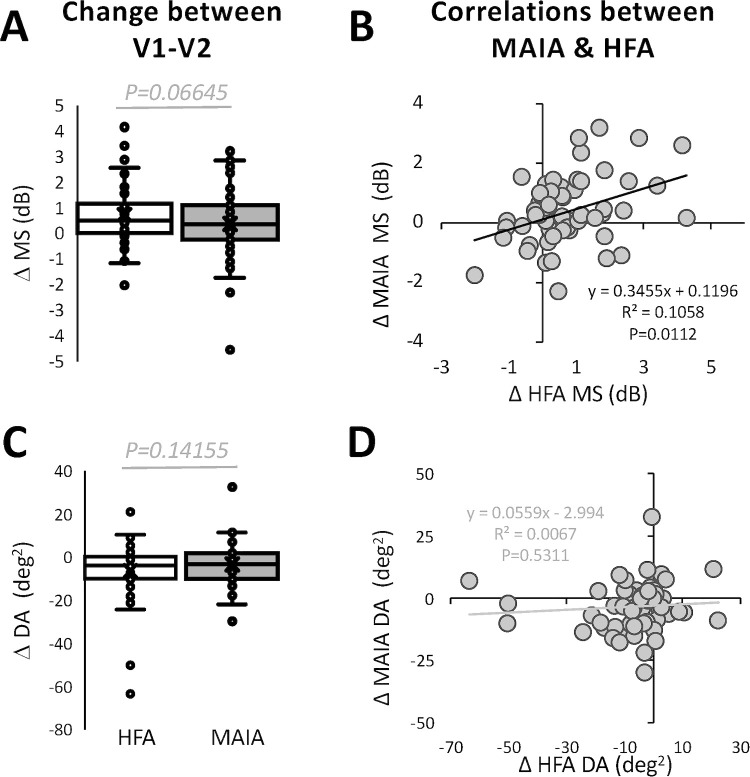
Change in HFA and MAIA outputs across visits. (A) Box and whisker plot of change in MS between visits 1 and 2, measured on the HFA and MAIA perimeters. Boxes denote the interquartile range (Q1 to Q3) with the line in the middle of the box denoting the median. Whiskers represent minimum and maximum values. Mean is indicated with an “X.” Significance test: two-tailed paired Student's *t* test, t_59_ = 1.87; 95% CI, ±0.50. (B) Linear regression analysis of change in HFA vs. MAIA MS, with individual circles representing individual patient data points. (C) Plot of change in HFA vs. MAIA deficit area (DA). All conventions as in (A). Significance test: two-tailed paired Student's *t* test, t_59_ = 1.87; 95% CI, ±0.50. (D) Linear regression analysis of change in HFA vs. MAIA deficit areas (DA). All conventions as in (B).

Across the tested field of view, the change in HFA and MAIA MS from visit 1 to 2 was similar (Fig. 5A) and significantly correlated (Fig. 5B). However, the correlation was weaker (R^2^ = 0.11), than those between HFA and MAIA MS within each visit (Fig. 4A). The shallow slope of the linear regression fit indicated that for every 1 dB change in HFA MS, there was only 0.35 dB of change in MAIA MS. Likely related to this, although the magnitude of change in the deficit area on the two machines was also similar (Fig. 5C), they were no longer correlated (Fig. 5D).

### HFA and MAIA Reliability Metrics Across Visits

**Figure 6.**
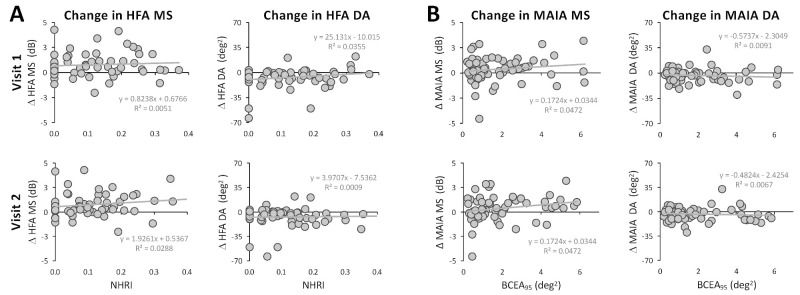
Test reliability metrics at visits 1 and 2 do not correlate with changes in HFA or MAIA outputs. (A) Linear regression analyses of HFA MS and deficit area (DA) with the NHRI computed at each visit. No significant relationships were observed. (B) Linear regression analyses of MAIA MS and DA plotted against the BCEA_95_ at each study visit. No significant relationships were observed for either MAIA output measure.

Although strict reliability criteria were used to include patients' visual fields in the present analysis, participants still exhibited variance within the allowable threshold of 20% false positives, false negatives, and fixation losses (Table 2). As such, we asked whether this variance explained the low or lack of correlation between HFA and MAIA MS and deficit area change, respectively. None of the HFA reliability metrics, nor the MAIA BCEA_95_, changed significantly between visits 1 and 2 (Table 2). Furthermore, change in the HFA MS and deficit area failed to correlate with NHRI (Fig. 6A); change in the MAIA MS and deficit area also failed to correlate with BCEA_95_ at either visit (Fig. 6B). Consistent with these observations, we saw no significant correlations between HFA MS, deficit area, and individual HFA reliability metrics (Supplementary Fig. S5), or between the MAIA MS, deficit area, and BCEA_95_ at either visit (Supplementary Fig. S6).

**Table 2.** Test Reliability Metrics Across Visits

				Paired *t* Tests Comparing Visits 1 and 2		
	Visit 1	Visit 2	Change Visit 1 – Visit 2	*t*	*P* Value	95% CI
HVF fixation losses	2.6 ± 2.8 %	3.1 ± 2.8 %	0.5 ± 3.6 %	t_55_= −0.27	0.49	±0.21
	(0% to 11.0 %)	(0% to 10.9 %)	(−6.5% to 6.3 %)			
HVF false positives	1.6 ± 3.1 %	1.7 ± 3.7 %	0.1 ± 4.4 %	t_55_= −1.06	0.29	±0.97
	(0% to 14.3 %)	(0% to 19.6 %)	(−14.3% to 19.6)			
HVF false negatives	2.9 ± 3.8 %	2.6 ± 4.5 %	−0.34 ± 6.1 %	t_55_= 0.22	0.83	±1.17
	(0% to 11.8 %)	(0% to 18.8 %)	(−11.8% to 18.8 %)			
HVF short-term	1.7 ± 0.7 dB	1.8 ± 0.6 dB	0.07 ± 0.8 dB	t_55_= −0.39	0.68	±1.62
fluctuations	(0.5 to 3.2 dB)	(0.7 to 4.2 dB)	(−1.5 to 2.6 dB)			
MAIA BCEA_95_	1.8 ± 1.6 deg^2^	1.9 ± 1.6 deg^2^	0.1 ± 1.4 deg^2^	t_59_= −0.54	0.59	±0.36
	(0.3 to 6.2 deg^2^)	(0.2 to 5.9 deg^2^)	(−4.5 to 3.1 deg^2^)			

HVF, Humphrey visual field.

Values in the first three columns are means ± standard deviations [range].

## Discussion

The present analysis compared visual deficit parameters measured by two automated perimeters after stroke-induced occipital cortex damage, with strict inclusion criteria for both patients and test performance. Our first goal was to determine whether the MAIA and HFA provided equivalent estimates of homonymous visual field defects in terms of their area and MS across the central ±10° of the visual field at isolated timepoints. Our second goal was to determine whether the two devices exhibited similar sensitivity to change in deficit size and MS.

### Basic Comparisons

In normally sighted, healthy participants, results from the HFA and MAIA perimeters correlate well.^32^ An excellent correlation between HFA MD and MAIA MS^32^^,^^33^ and macular HFA MS and MAIA MS^34^ was also reported in patients with glaucoma. We now show that HFA and MAIA outputs are generally well-correlated in patients with homonymous visual field defects from occipital strokes, although MAIA threshold values (represented here by MS) are systematically lower than those measured side by side using the HFA. Predictably, deficit areas defined by a unitary criterion of less than 10 dB^31^ were larger in the MAIA than HFA. This can be explained, at least in part, by the differing maximum stimulus brightness (10,000 asb in the HFA; 1000 asb in the MAIA) and the photopic (HFA) vs. mesopic (MAIA) testing paradigms. Thus, the 10-dB criterion for defining blindness, which was designed for use in photopic tests like the HFA, may in fact be inappropriate for use with the mesopic MAIA perimeter. Indeed, a nonlinear relationship exists between mesopic and photopic contrast sensitivity in normal observers, with good mesopic performance being predictive of good photopic performance, but not necessarily the opposite.^35^ In patients with glaucoma, the disparity between photopic and mesopic performance becomes even greater.^35^ Interestingly, a loss of retinal ganglion cells is common to both CB and glaucoma; in CB patients, trans-synaptic retrograde degeneration initiated by damage to primary visual cortex^36^^–^^39^ is the main cause. Thus, one might predict that, just like patients with glaucoma, those with CB would be likely to exhibit larger differences than normal between photopic and mesopic contrast sensitivity.

### HFA and MAIA Perimeters Differ in Their Ability to Measure Visual Field Change

Our next question explored whether fundamental differences between the two perimeters led to differences in their ability to measure change in performance, either as a function of interventions^1^^,^^19^^–^^25^^,^^40^ or spontaneously over time.^41^^–^^44^ We now report, for the first time, that although HFA and MAIA perimeters detected similar within-patient changes in performance between visits, the strength of correlation between HFA- and MAIA-derived changes was weak. In a few cases, large discrepancies were seen (e.g., one device reporting worsening sensitivity and the other improvement for the same patient). However, although this was true for both outcome measures examined here, it seemed to be much worse for deficit area than for MS, suggesting a key contribution of the impaired hemifield, or how it is defined in the two perimeters.

As a first step toward understanding this outcome, we first examined test reliability scores and asked if—despite staying within the acceptable range for inclusion—they changed significantly or unevenly in the two devices between visits. The simple answer was no, even when scores were combined into a single reliability index (NHRI) for the HFA. Small differences in reliability between participants could reflect differences in approach—for example, a higher rate of false-positive responses, while staying below our 20% cut-off, would suggest a propensity to over-respond to stimuli, leading to artificially high reported sensitivity in the blindfield.^26^^,^^45^^,^^46^ Likewise, a higher rate of false negatives could indicate an overly conservative response approach, leading to artificially lowered sensitivities.^26^^,^^46^^,^^47^ Here, we did not observe differences in reliability scores between machines or changes in these scores between visits, and there was no relationship between individual visit MS or deficit area and reliability scores on either machine. The patients’ reliability metrics at visits 1 or 2 also did not predict performance changes measured between visits on either machine. Although fixation losses have been reported to decrease reliability of HFA examinations, one study showed negligible impact on predicted MD in patients with glaucoma.^46^ This is not to discount the impact of even the smallest fixational eye movements on perimetric reliability; it has been argued that prior research may have overestimated the beneficial impact of computer-based light detection training in CB by not controlling fixational eye movements during perimetry-based assessment of visual field change.^15^^,^^16^ Here, contrary to what might be predicted if patients’ visual deficits shrank because they learned to move their eyes and cheat (as in Reinhard et al.^15^), none of the fixation-related tests scores (% fixation losses on the HFA, BCEA_95_ on the MAIA) predicted the magnitude of MS or deficit area changes (including those denoting improvement in the visual deficit) seen with either device. Thus, we conclude that the decorrelation observed for visual field change between the MAIA and HFA is not attributable to differences in test validity owing to learning to cheat or adopting other test performance strategies detectable on either perimeter. Instead, our prime hypothesis for explaining the greater rate of change in HFA performance relative to the MAIA is that the training intervention (administered to most of the patients in this cohort) likely caused improved thresholds at several impaired hemifield locations. As was shown by our breakdown analysis of the intact vs. impaired hemifields, the HFA systematically reports larger MS values than the MAIA, and the differences are much greater for the intact than the impaired hemifield. These data predict that the HFA should report larger MS changes when sensitivity reaches near-intact field values in a given impaired hemifield. Indeed, it has been suggested previously that visual training in CB may offer differing levels of benefit when measured with different perimeters.^17^ However, as discussed elsewhere in this article, it is also possible that a key reason for the outcome observed presently is the poorer detection sensitivity of CB patients when performing mesopic perimetry.

### Considerations for Choosing the HFA vs. MAIA in CB

The HFA is considered by many to be the clinical standard for measuring visual field defects in CB.^8^ The introduction of scanning laser ophthalmoscopy into perimetry in the 1980s^48^ provided an alternative for measuring vision and “blindness.” The present study adds to the literature contrasting performance of different perimeters by highlighting differences in how these systems assess vision in CB. The HFA is most often used in glaucoma,^26^ and the MAIA was purposed to identify macular diseases,^30^ including diabetic retinopathy and macular degeneration. Thus, neither device was designed to assess visual impairment in patients after occipital stroke. Our analysis shows that, at given timepoints, assuming high-quality test performance by the patient, the HFA and MAIA yield similar qualitative and quantitative estimates of global visual sensitivity, and of the location of the visual field defect. However, there are also systematic differences between the two perimeters, with the HFA reporting greater overall visual sensitivity and smaller deficit areas than the MAIA, when using a unitary definition of blindness. These differences may not be critical for simply diagnosing the deficit, but they could matter when seeking to detect small and/or localized changes in performance that occur spontaneously or after therapeutic interventions. Here, both devices detected changes in performance in patients who had undergone vision restoration (anticipated to yield improved MS and decreased deficit area^18^) or no training (anticipated to yield either spontaneous improvement, no change, or worsening in test performance^18^). HFA and MAIA changes were correlated positively in terms of MS, but not for deficit area. We posit that the lack of correlation in deficit area arose in part from our 10-dB threshold for blindness, a definition designed for use in Humphrey perimetry.^31^ In addition, the larger range of luminances and the finer steps in which they are presented by the HFA theoretically impart this perimeter with greater sensitivity to change—either improvement or worsening—relative to the MAIA. Finally, we note that our results should not be interpreted to claim that one device better represents a patient's vision than the other. If administered correctly, with reliability metrics adhered to, any perimetric test can provide a useful estimate of a patient's vision.

## Conclusions

The HFA and MAIA perimeters yield similar but systematically different visual sensitivity and deficit area measurements at individual timepoints after occipital stroke, which impact their sensitivity to changes over time and as a function of therapeutic interventions. These differences are likely attributable to the distinct test conditions of the devices: the staircases used for threshold detection differ, the machines present an increment detection vs. a light detection task, and the HFA tests under photopic conditions, which may be easier for CB patients than the mesopic conditions of the MAIA. Both machines remain reliable and viable options for assessing CB visual deficits and, critically, changes in those deficits after visual training. All in all, the pros and cons of each machine, including testing time, conditions, and fixation controls, should be weighed when assessing each new patient with CB, with a final recommendation that different criteria defining blindness should be established for each instrument.

## Supplementary Material

Supplement 1
