# Supplementary material for: MAIA and Humphrey Perimetry Differ in Their Estimation of Homonymous Visual Field Defects
Source: Transl Vis Sci Technol. 2024 Nov 14;13(11):15. doi: 10.1167/tvst.13.11.15 (PMC11572756; doi:10.1167/tvst.13.11.15)
Supplement: Supplement 1 [file tvst-13-11-15_s001.pdf]

Supplementary Materials

Redmond et al.

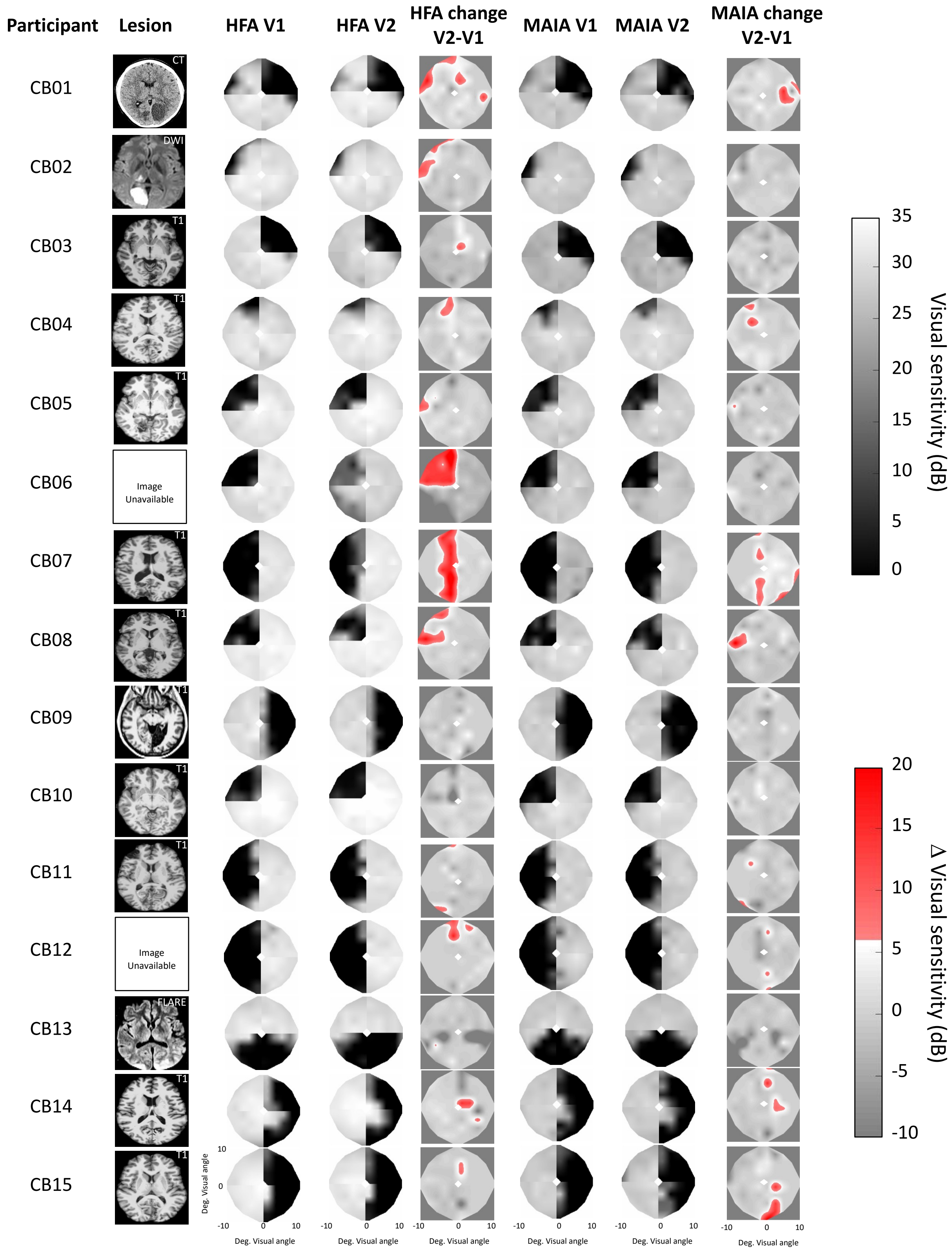

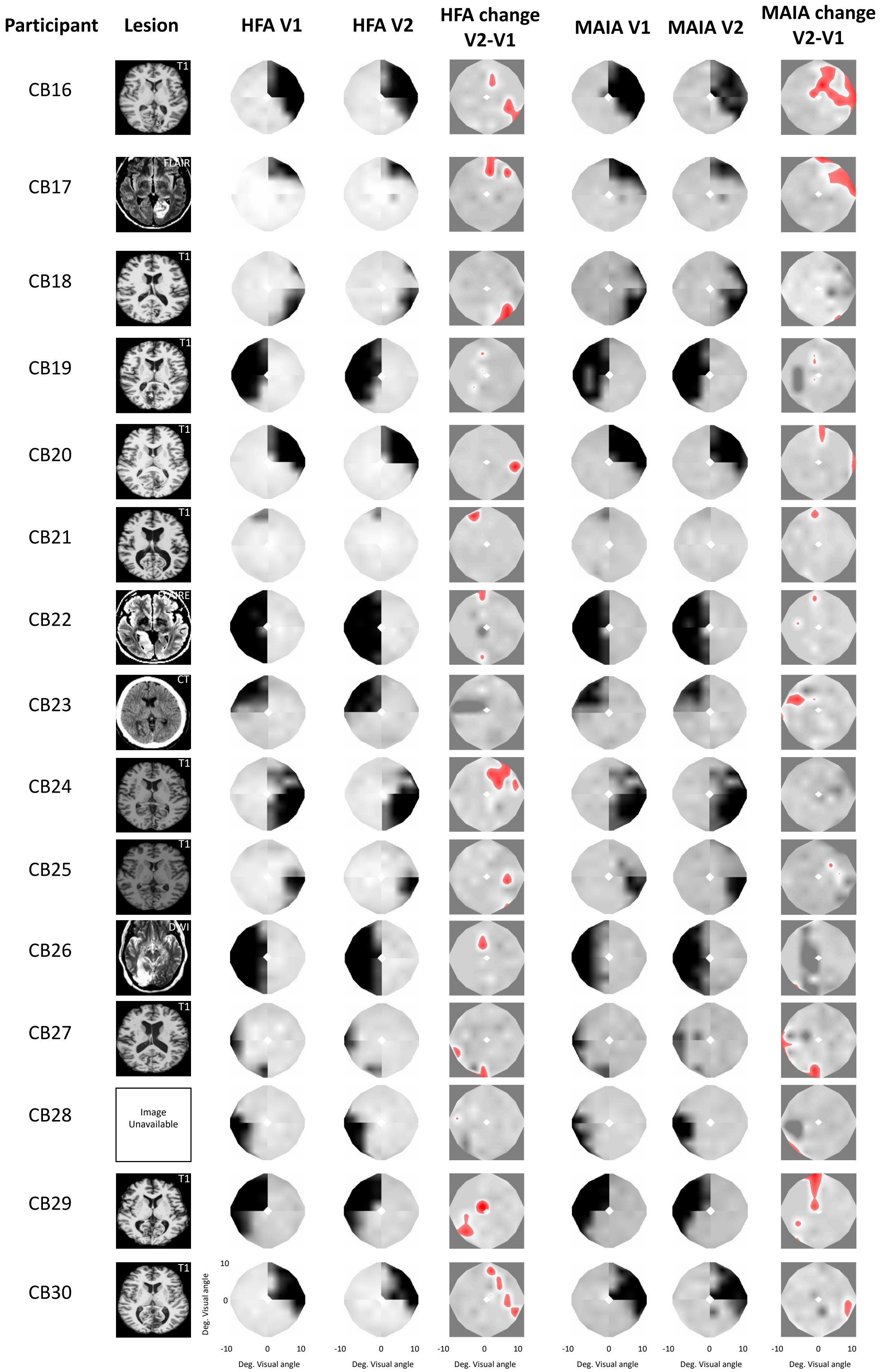

Participant
Lesion
HFA V1
HFA V2
HFA change V2-V1
MAIA V1
MAIA V2
MAIA change V2-V1
CB16
CB17
CB18
CB19
CB20
CB21
CB22
CB23
CB24
CB25
CB26
CB27
CB28
CB29
CB30
T1
FLAIR
CT
DWI
Image Unavailable
Deg. Visual angle
-10
0
10

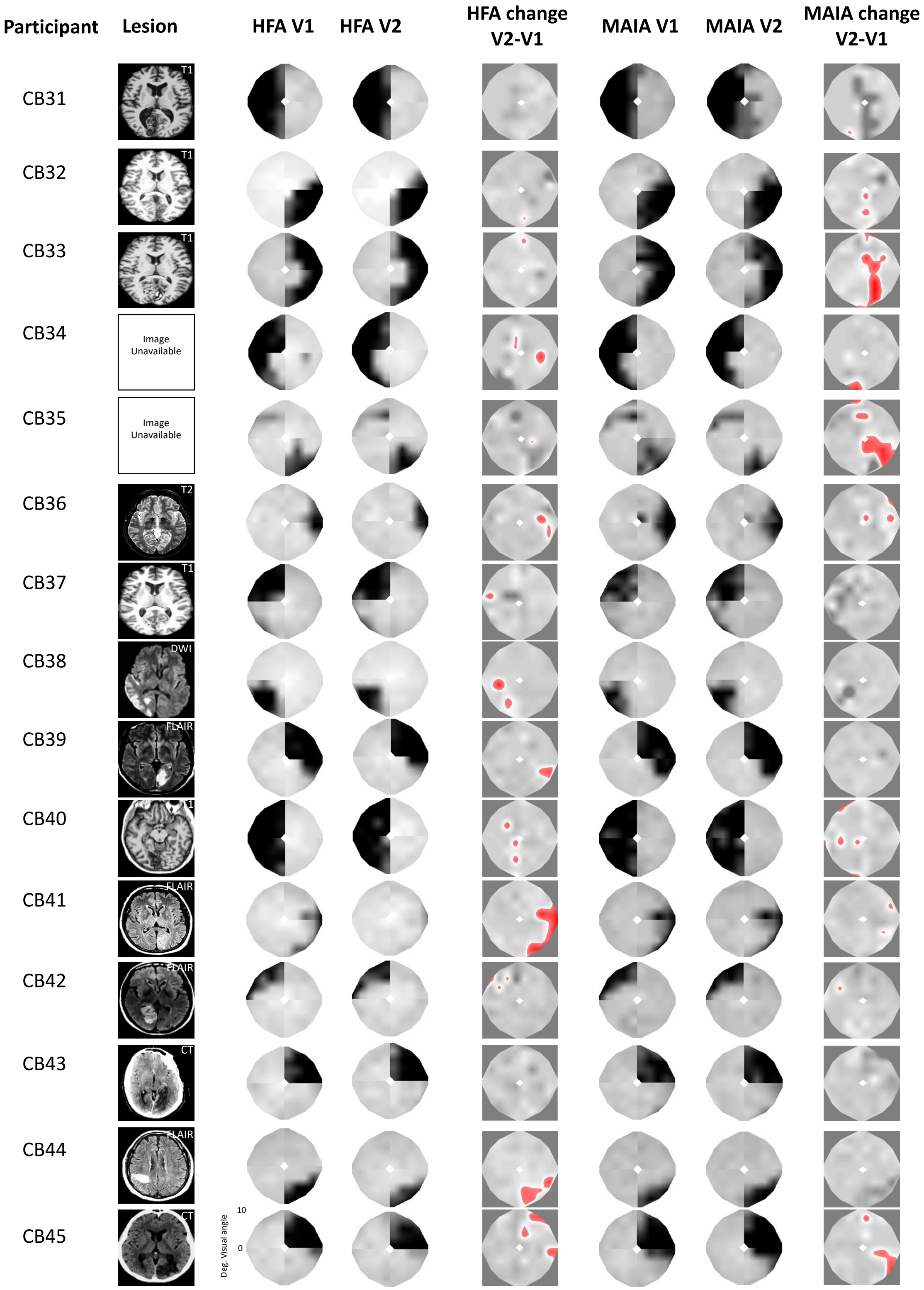
Participant
Lesion
HFA V1
HFA V2
HFA change V2-V1
MAIA V1
MAIA V2
MAIA change V2-V1
CB31
CB32
CB33
CB34
CB35
CB36
CB37
CB38
CB39
CB40
CB41
CB42
CB43
CB44
CB45
T1
T1
T1
Image Unavailable
Image Unavailable
T2
T1
DWI
FLAIR
T1
FLAIR
FLAIR
CT
FLAIR
CT
10
0
Deg. Visual angle

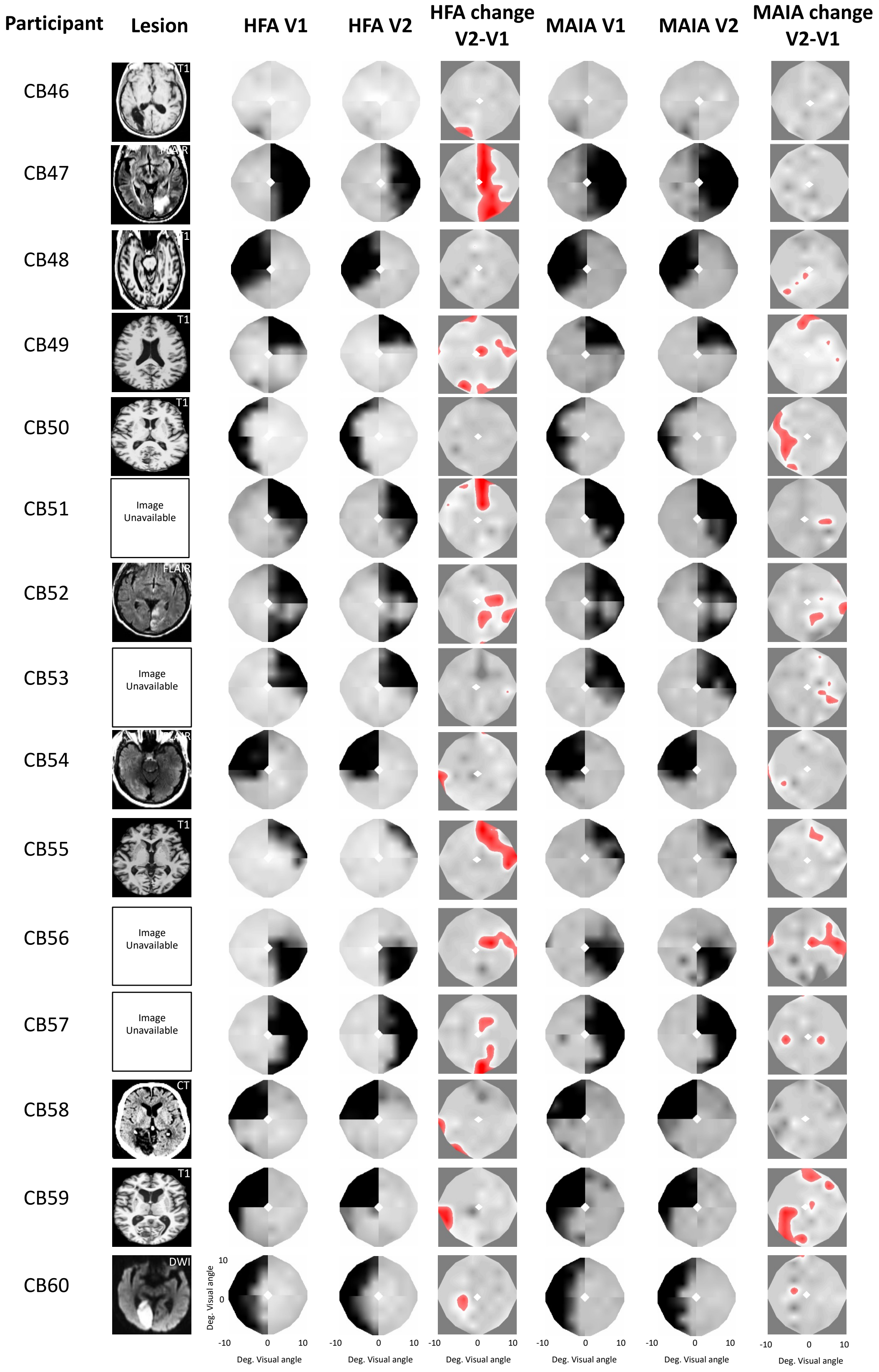

Supplementary Figure S1. The first column shows MRI (magnetic resonance imaging) or CT (computed tomography) pictures for each patient, in standard radiologic convention (with right brain hemispheres on image left). DWI = diffusion weighted imaging, FLAIR = fluid attenuated inverse recovery. Columns 2-4 show HFA interpolated maps at Visit 1, Visit 2 and change from Visit 1-2. Columns 5-7 show MAIA interpolated maps at Visit 1, Visit 2, and change from Visit 1-2. The grey scale represents raw sensitivity values for maps shown in columns 2, 3, 5 and 6. The color scale denotes changes in sensitivity in the columns 4 and 7, thresholded so that areas of red indicate changes ≥6dB.

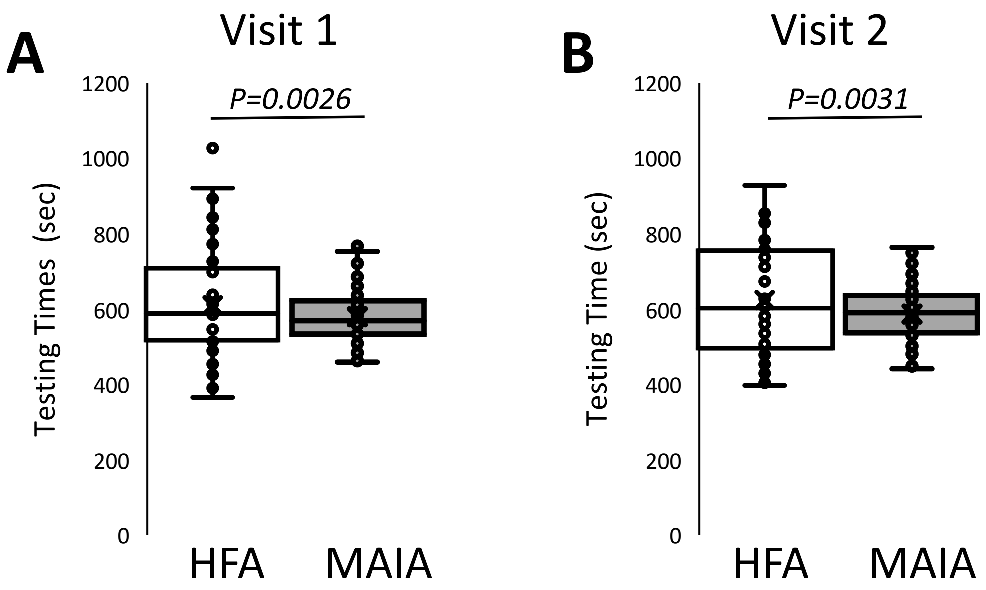

Supplementary Figure S2. Comparison of monocular testing time for HFA and MAIA perimeters. A. Box and whisker plots at Visits 1. Boxes denote the interquartile range (Q1 to Q3) with the horizontal line in the middle of the box denoting the median. Whiskers at each end represent the minimum and maximum values. Means are indicated with "X". Paired Student's t-tests showed a significant difference in duration between the devices (t_{111}=3.04, CI_{95}=±0.41) **B.** Visit 2 HFA and MAIA test time comparison, with paired Student's t-tests showing a significant difference (t_{111}=3.02, CI_{95}=±0.44).

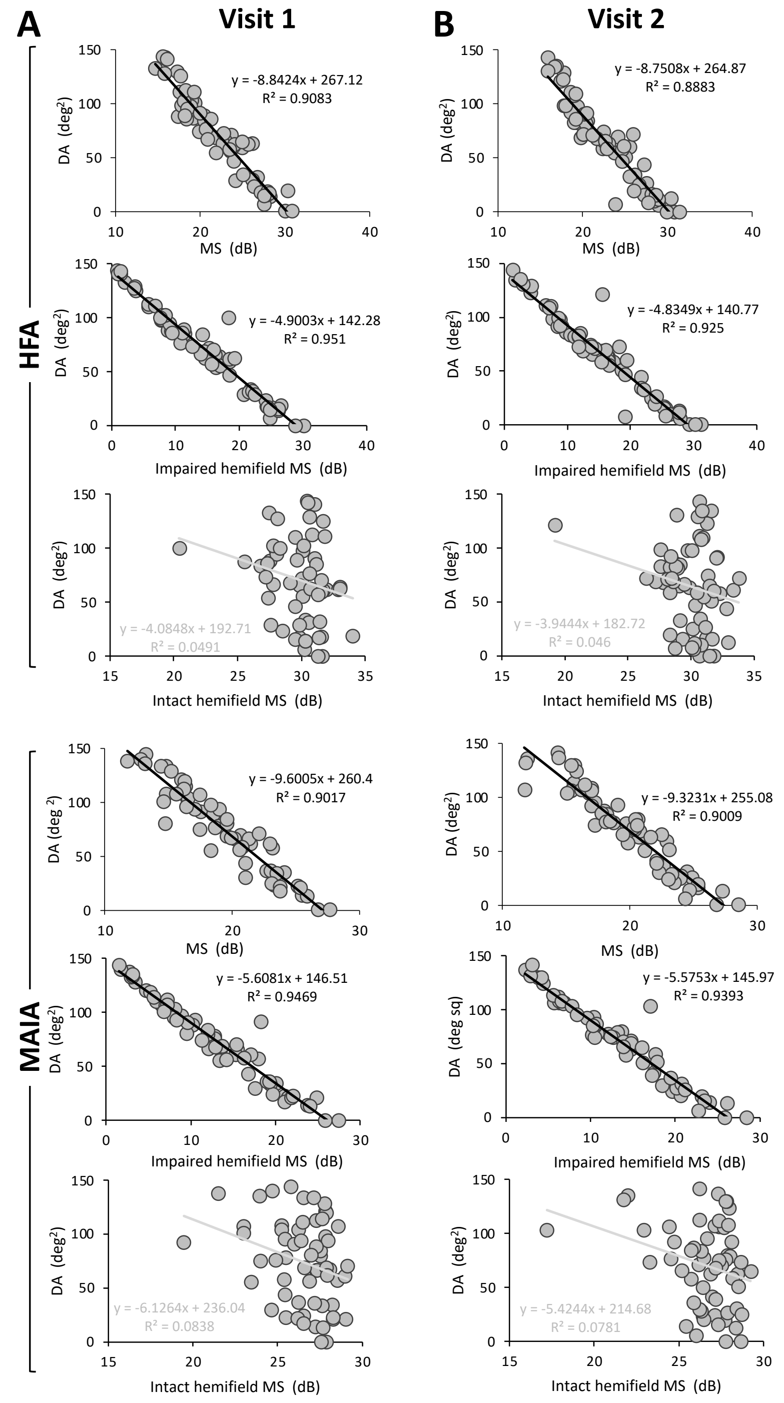

Supplementary Figure S3. Significant correlation in both HFA and MAIA between deficit area (DA) and whole-filed or impaired hemifield MS - not intact hemifield MS. A. Linear regression analysis of HFA and MAIA metrics at Visit 1, fitted to data from individual patients. **B**. Same data as in A for Visit 2. Black regression lines denote statistical significance; grey regression lines denote lack of statistical significance.

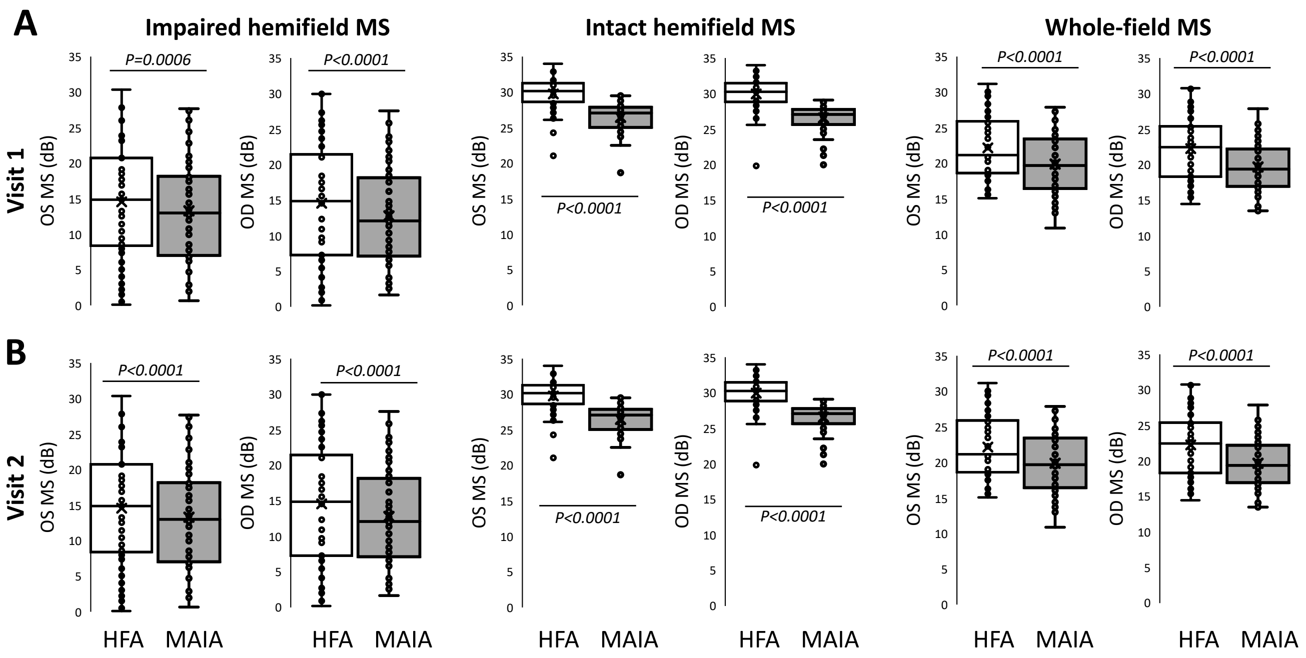

Supplementary Figure S4. Monocular HFA and MAIA-derived outcome measures at each visit. Box and whisker plots of monocular mean sensitivity (MS) and deficit area (DA) at Visits 1 **(A)** and Visit 2 **(B)** on each machine. Boxes denote the interquartile range (Q1 to Q3). Horizontal lines in the middle of each box denote the median. Whiskers represent the minimum and maximum values. Means are indicated with "X" in each box. Paired Student's t-tests show significant differences in all comparisons, a result recapitulated when averaging data between eyes for each patient (see Fig. 3 A-C).

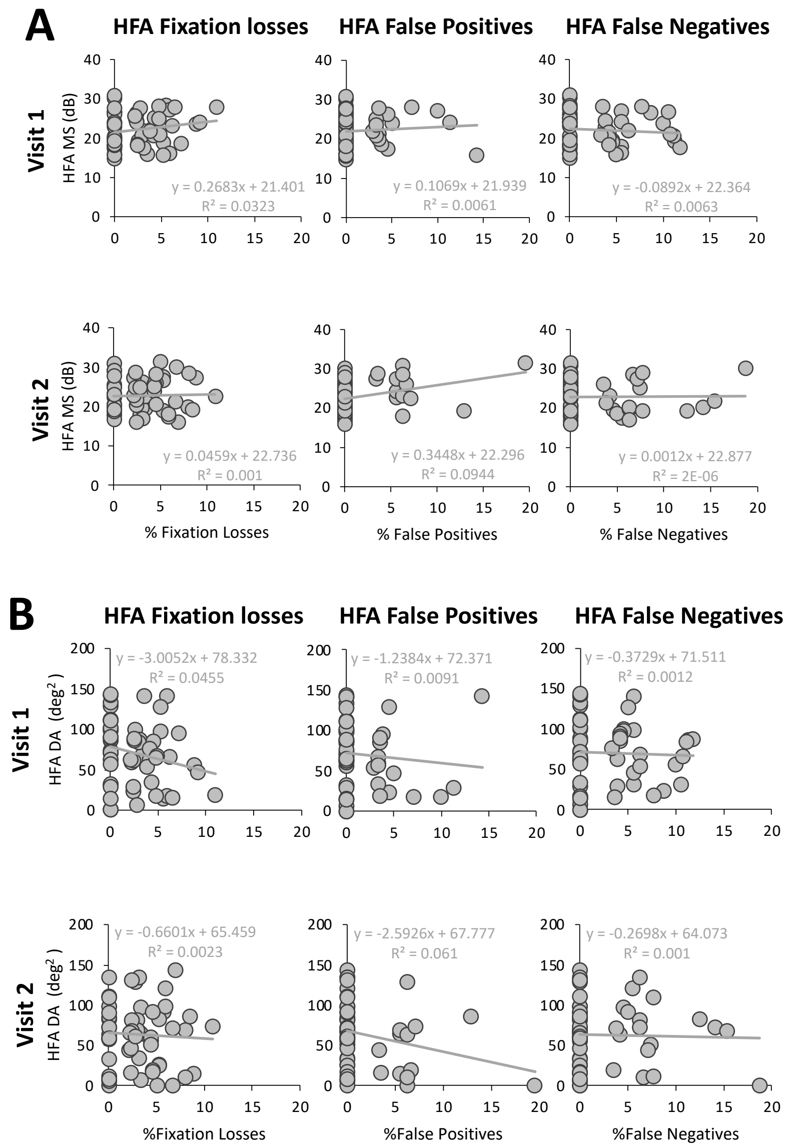

Supplementary Figure S5. Individual HFA reliability metrics do not correlate with performance at either visit. A. Linear regression analyses of HFA MS with fixation losses, false positives and false negatives at each visit. No significant relationships were observed. **B.** Identical regression analyses as in A for HFA DA. Once again, no significant relationships were observed (grey regression lines).

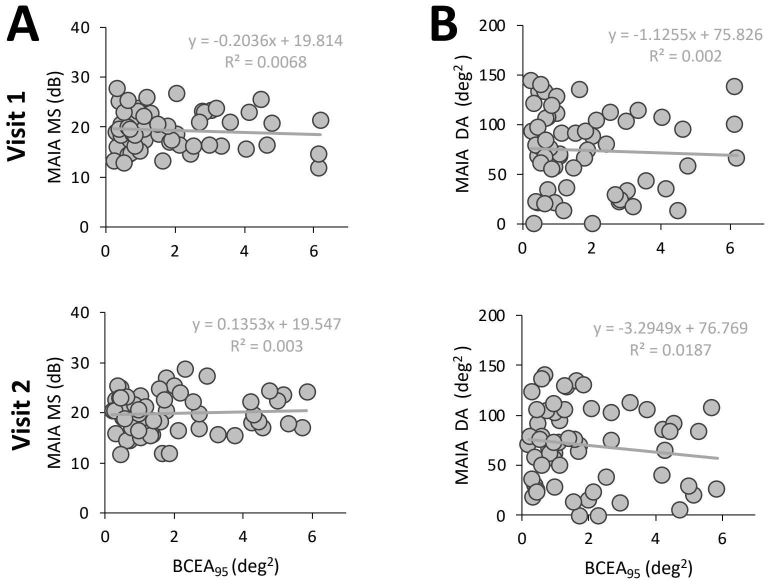

Supplementary Figure S6. MAIA BCEA does not correlate with performance at individual visits. A. Linear regression analyses of MAIA MS with $BCEA_{95}$ at each visit. No significant relationships were observed. **B.** Identical regression analyses as in A for MAIA deficit area (DA). Once again, no significant relationships were observed (grey regression lines).
